# The effects of 5-hydroxytryptophan on attention and central serotonin neurochemistry in the rhesus macaque

**DOI:** 10.1038/s41386-017-0003-7

**Published:** 2018-01-30

**Authors:** Hannah Weinberg-Wolf, Nicholas A. Fagan, George M. Anderson, Marios Tringides, Olga Dal Monte, Steve W. C. Chang

**Affiliations:** 1 Department of Psychology, Yale University, New Haven, CT 06520 USA; 2 Child Study Center, Yale University School of Medicine, New Haven, CT 06510 USA; 3 Department of Neuroscience, Yale University School of Medicine, New Haven, CT 06510 USA; 4 Kavli Institute for Neuroscience, Yale University School of Medicine, New Haven, CT 06510 USA

## Abstract

Psychiatric disorders, particularly depression and anxiety, are often associated with impaired serotonergic function. However, serotonergic interventions yield inconsistent effects on behavioral impairments. To better understand serotonin’s role in these pathologies, we investigated the role of serotonin in a behavior frequently impaired in depression and anxiety, attention. In this study, we used a quantitative, repeated, within-subject, design to test how L-5-hydroxytryptophan (5-HTP), the immediate serotonin precursor, modulates central serotoninergic function and attention in macaques. We observed that intramuscular 5-HTP administration increased cisternal cerebrospinal fluid (CSF) 5-HTP and serotonin. In addition, individuals’ baseline looking duration, during saline sessions, predicted the direction and magnitude in which 5-HTP modulated attention. We found that 5-HTP decreased looking duration in animals with high baseline attention, but increased looking duration in low baseline attention animals. Furthermore, individual differences in 5-HTP’s effects were also reflected in how engaged individuals were in the task and how they allocated attention to salient facial features—the eyes and mouth—of stimulus animals. However, 5-HTP constricted pupil size in all animals, suggesting that the bi-directional effects of 5-HTP cannot be explained by serotonin-mediated changes in autonomic arousal. Critically, high and low baseline attention animals exhibited different baseline CSF concentrations of 5-HTP and serotonin, an index of extracellular functionally active serotonin. Thus, our results suggest that baseline central serotonergic functioning may underlie and predict variation in serotonin’s effects on cognitive operation. Our findings may help inform serotonin’s role in psychopathology and help clinicians predict how serotonergic interventions will influence pathologies.

## Introduction

For decades, researchers have studied the relationship between the serotonergic system and depression and anxiety, neuropsychiatric disorders that are often comorbid [1–5]. Individuals with depression and anxiety typically experience impaired executive function and emotional cognition, symptoms that are generally studied by examining disruptions in attention and the recognition of emotions [6–10]. Previous work has increased central serotonergic functioning, using selective serotonin reuptake inhibitors (SSRIs) or tryptophan loading, to improve how patients attend to, and process, information in their environments [3, 11–14]. Conversely, reducing circulating levels of the serotonin precursor tryptophan in healthy humans via Acute Tryptophan Depletion (ATD) impairs emotion recognition and information processing, mimicking aspects of depression and anxiety symptomatology [11, 15–17].

Serotonergic function has also been linked to competent cognition and mood regulation in human and non-human primates in non-clinical contexts [18, 19]. In vervet monkeys, enhancing serotonergic function with a diet chronically high in tryptophan led to an increase in dominance status, a decrease in aggression, and an increase in affiliative and social bonding behaviors [20]. Conversely, impaired serotonergic functioning, assessed via cisternal cerebrospinal fluid (CSF) concentrations of the serotonin metabolite 5-hydroxyindoleacetic acid (5-HIAA), is correlated with low dominance rank, poor impulse control, impaired social functioning, extreme aggression, severe wounding, and even mortality in rhesus macaques [21–29]. However, the gross behavioral measures derived from observational data do not provide the resolution needed to determine if serotonin’s effects on behavior are driven by changes in how individuals allocate attention to behaviorally-relevant stimuli.

The majority of previous studies examining the relationship between serotonin and attention in humans have used between-subject designs, or, when using within-subject designs, collected only one session of data per condition for each subject [11]. Given that CSF collection requires invasive methods, most studies were unable to examine how, and if, serotonergic manipulations modulate central serotonergic function differently across subjects. Central serotonergic function was examined in the present study by measuring CSF levels of L-5-hydroxytryptophan (5-HTP), serotonin (5-HT), and 5-HIAA across subjects. Perhaps as a consequence, past studies have reported inconsistent and sometimes difficult to interpret effects of serotonin manipulations on attention [11]. Thus, the causal link between serotonin and impaired attention remains elusive, as do the underlying biological mechanisms mediating these effects.

In the current study, we employed a quantitative and controlled within-subject design to clarify the causal and mechanistic relationship between serotonin and attention in rhesus macaques. We tested whether administering intramuscular (i.m.) injections of the serotonin precursor 5-HTP, which human and rodent literature suggests is an effective method to acutely increase central concentrations of serotonin [30–33], would modulate rhesus macaques’ attention to social and non-social images using a well-established free-viewing task [34, 35]. In addition, we measured cisternal CSF concentrations of serotonin, along with its precursors (tryptophan, 5-HTP), and its principle metabolite, 5-HIAA, to assess serotonin metabolism and obtain an index of extracellular, functionally-active serotonin in the brain. Specifically, we examined how 20 mg/kg or 40 mg/kg 5-HTP administrations, compared to a saline control, modulated central concentrations of 5-HTP and affected serotonin neurochemistry and related these acute changes to modulations in looking behavior measured stably by collecting eight sessions of data per drug condition, per subject.

## Materials and methods

### Test subjects

Six adult (five male and one female; aged 5–8 years (5.5 ± 1.22)) rhesus monkeys (*Macaca mulatta*) served as subjects. Subjects weighed between 6.8 and 16.7 kg throughout the duration of the study. Subjects were housed with a single pair (*n* = 3) or in triads (*n* = 3), kept on a 12-h light/dark cycle, had unrestricted access to food 24-h a day, and controlled access to fluid during testing. All procedures were reviewed and approved by the Yale University Institutional Animal Care and Use Committee.

### Experimental design

**Fig. 1:**
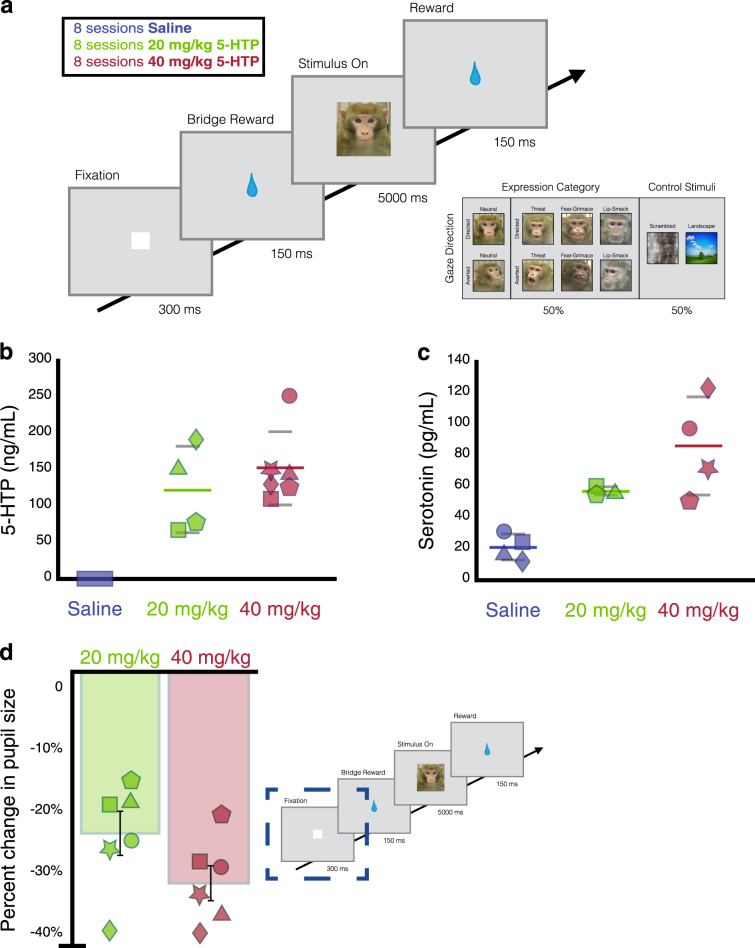
Behavioral task, CSF concentrations of 5-HTP and serotonin, and effect of 5-HTP on autonomic arousal. **a** Behavioral task and stimuli. Example social and non-social control images are seen on the right. **b** CSF concentration of 5-HTP. The central concentration of 5-HTP after i.m. injection of saline (blue), 20 mg/kg 5-HTP (green), or 40 mg/kg 5-HTP (red). **c** CSF concentration of serotonin. The central concentration of serotonin after i.m. injection of saline (blue), 20 mg/kg 5-HTP (green), or 40 mg/kg 5-HTP (red). **d** 5-HTP constricts the pupil. The percent change from saline in the size of pupil during the fixation period of trials during 20 mg/kg 5-HTP (green) and 40 mg/kg 5-HTP (red) sessions. In **b**–**c**, the average CSF concentration per dose is represented by a colored line, and grey lines represent the standard error. In **b**–**d**, Each shape represents an individual subject’s data

Subjects viewed stimulus images in a testing room, alone, on an LCD computer monitor positioned 36 cm away from the subject and that spanned 40 × 30 degrees of visual angle with a temporal resolution of 2 ms. Subjects viewed unaltered conspecific face stimuli taken from a large library of static monkey face images described by Gothard et al. [36]. Rhesus macaques rely on a set of highly stereotyped, species-specific social signals to maintain dominance [37]. Open-mouthed threat faces are used to communicate dominance or intent to maintain control over a resource, while the bared-teeth fear grimace is used to communicate fear and submission [38]. Rhesus macaques also use lip-smacks as an affiliative gesture to diffuse aggression [39]. Stimulus monkeys displayed one of these three standard facial expressions, or a neutral expression, with either direct or averted gaze (Fig. 1a). Subjects had neither seen nor interacted with any of the monkeys depicted in the images, so the 51 unique identities whose faces we included were unfamiliar and novel to the subjects.

We divided our total amount of images into four unique sets. Each set consisted of 24 conspecific faces per each of the eight image categories. This resulted in 192 unique faces per set. Each image set also contained equal numbers (96 images per set) of scrambled faces and landscape images so that the total images viewed during any sessions consisted of 50% faces (192 images), 25% scrambled faces (96 images), and 25% landscapes (96 images). The subjects completed two sessions of data collection per day. We collected 1 day of data per set of images for each drug dose. This means that we collected 4 days of data per drug dose, each with a unique set of images. Because we collected two separate sessions of data per day, this resulted in eight sessions total per drug dose per subject. To preclude order effects, we counterbalanced the order in which we selected image sets while ensuring that subjects were never exposed to the same set of images during two sessions in a row. Within a single session of data collection, we also counterbalanced and randomized the order of image presentation to preclude order effects.

### Pharmacological methods

5-HTP has several advantages, compared to SSRIs or tryptophan loading, when enhancing serotonergic function in acute studies. 5-HTP is the immediate precursor to serotonin and is administered in smaller doses than tryptophan and for both reasons can be presumed to produce fewer collateral effects on brain catecholamine, trace amine, and kynurenine pathway neurochemistry [31, 33, 40]. 5-HTP has been demonstrated to be active for 1–4 h after i.m. injection, allowing finer temporal control than tryptophan loading [30, 32]. Finally, although SSRIs appear to produce rapid acute increases in central extracellular serotonin [41], it has been suggested that some of their effects on serotonergic function and on behavior require chronic administration [42].

All pharmacological treatments were administered (between 12:30 and 14:30 daily) intramuscularly (i.m.) exactly one hour before testing onset. Administered volume was consistently between 1.0 and 2.0 mL depending on the weight of the subject. l-5-HTP (Sigma) was suspended in sterile water and given at 20 mg/kg or 40 mg/kg doses. Each subject received four injections of saline, four injections of 20 mg/kg 5-HTP, and four injections of 40 mg/kg. Because subjects completed two sessions of the task on each day, one that began 1 h after injection and one that began 1 h and 50 min after injection, this resulted in eight sessions of data per drug dose per animal. Drug doses were delivered on strictly alternating days, with no 5-HTP doses being delivered 2 days in a row. Vehicle injections consisted of equal volumes of sterile saline. Doses for 5-HTP were selected on the basis of previous studies in rodents and human subjects [31, 43] showing that 5-HTP doses less than 20 mg/kg do not produce discernable behavioral effects. In addition, previous studies suggest that doses greater than 60 mg/kg can inadvertently increase circulating catecholamines by displacing catecholamines from storage granules, thereby temporarily enhancing postsynaptic catecholaminergic stimulation [41].

### CSF sample collection and assays

To determine whether i.m. 5-HTP crossed the blood brain barrier in rhesus macaques, to test if injections increased central levels of 5-HTP and serotonin, and to determine how variation in serotonergic function related to 5-HTP’s effects on behavior, we sampled CSF from each subject after receiving an i.m. injection of saline, 20 mg/kg 5-HTP and 40 mg/kg 5-HTP with a minimum of 2 weeks between each collection date. We counterbalanced and randomized subjects order of CSF sampling between saline, 20 mg/kg 5-HTP and 40 mg/kg 5-HTP. Each CSF draw occurred one-hour post injection, the same time after injection that animals began data collection daily. A complete set of CSF draws, one per each of the three drug conditions, was carried out in four out of six subjects. CSF was not collected from subject 2 at 20 mg/kg 5-HTP and from subject 5 at saline and 20 mg/kg due to complications with the procedure for these subjects.

Cisternal CSF was assessed with cervical punctures, which are preferred over lumbar punctures for accurately tracking concentrations of monoamines and monoamine metabolites in cortical and subcortical structures due to its greater proximity to the brain and its clearance from the spinal space [40, 44, 45]. Punctures targeted the cisterna magna through the atlanto-occipital membrane. Approximately 1.5 mL of CSF was drawn using a 24–27-gauge needle. Monkeys were first anesthetized with ketamine (3 mg/kg, i.m.) and dexdomitor (0.075 mg/kg, i.m.). To reverse anesthesia, we administered antisedan (0.075 mg/kg, i.m.) once the animal was returned to its cage after the draw. CSF was immediately labeled and frozen on dry ice before being transferred to a −70 degree Celsius freezer.

Supplementary Materials and Methods contain the details of the data analyses and other details on the experimental protocols.

## Results

We examined the effect of 5-HTP administrations on rhesus macaques’ (*n* = 6) natural viewing behavior to social (conspecific faces) and non-social (outdoor scenes and luminance matched scrambled faces) images while their eye positions were tracked with high spatial and temporal acuity (Fig. 1a). On separate days, we collected CSF samples 1 h after acute delivery of saline, 20 mg/kg, and 40 mg/kg 5-HTP to examine how 5-HTP influences central serotonergic function and to provide insight into the mechanism by which 5-HTP modulates attention.

### Exogenous 5-HTP increases CSF 5-HTP and serotonin concentrations and modulates autonomic arousal

We first tested if i.m. 5-HTP administrations increased central concentrations of 5-HTP and serotonin. CSF 5-HTP concentrations were higher after receiving 20 mg/kg (*P* = 0.03, Tukey) and 40 mg/kg 5-HTP (*P* < 0.01), compared to saline (Fig. 1b, *F*(2, 3) = 11.73, *P* < 0.01, ANOVA). 5-HTP administration also increased central serotonin, albeit more weakly (Fig. 1c, *F*(2, 3) = 9.46, *P* = 0.05). Posthoc tests indicate that this increase was driven by 40 mg/kg 5-HTP (20 mg/kg vs. saline: *P* = 0.13; 40 mg/kg vs. saline: *P* = 0.05). In addition, central concentrations of 5-HTP and serotonin are highly correlated with each other, indicating that increases in serotonin are proportional to the levels and dose of 5-HTP (*r* = 0.78, *P* < 0.01, Pearson’s correlation). We found no significant change in CSF concentrations of 5-HIAA due to exogenous 5-HTP (*F*(2, 3) = 1.29, *P* = 0.33). This is to be expected because the changes in CSF serotonin we observed were much smaller than the absolute levels of CSF 5-HIAA present biologically. Thus, the changes in 5-HIAA production due to 5-HTP administration would be diluted in the context of the much larger pool of CSF 5-HIAA. In addition, central concentrations of 5-HIAA were neither correlated with CSF 5-HTP (*r* = 0.50, *P* = 0.12) nor CSF serotonin (*r* = 0.24, *P* = 0.48). As expected, 5-HTP did not increase CSF concentrations of tryptophan (*F*(2, 3) = 1.72, *P* = 0.25), norepinephrine (*F*(2, 3) = 0.37, *P* = 0.72), the dopamine precursor tyrosine (*F*(2, 3) = 1.24, *P* = 0.35), or the dopamine metabolite homovanillic acid (HVA) (*F*(2, 3) = 1.20, *P* = 0.36). To see full pair-wise of CSF concentrations of monoamines, their precursors, and metabolites, see Supplementary Tables ST1, ST2, and ST3.

To assess physiological arousal, we quantified the size of the pupil during the 300 ms fixation period where only the luminance controlled white fixation square appeared on the screen. We found that 5-HTP did impact the size of the pupil (Fig. 1d, *F* (2, 3) = 46.35, *P* < 0.001, ANOVA). Subjects had a significantly more constricted pupil during 20 mg/kg (*P* < 0.001, Tukey) and 40 mg/kg 5-HTP sessions (*P* < 0.001) than saline sessions, indicating a consistent physiological effect of 5-HTP. Our CSF and pupil results indicate that i.m. 5-HTP administrations effectively increased central concentrations of 5-HTP and serotonin and impacted the parasympathetic system.

### Baseline behavior underlies the direction and magnitude of 5-HTP’s effects on attention

**Fig. 2:**
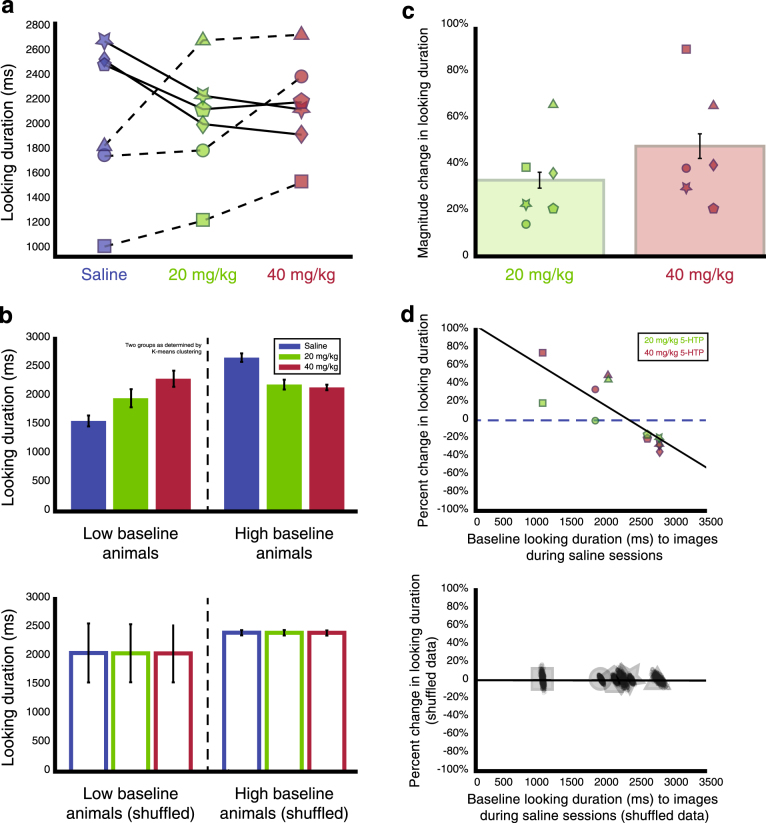
The direction and magnitude of 5-HTP’s effects on attention are rooted in baseline behavior. **a** 5-HTP causes a bi-directional change in looking duration. 20 mg/kg (green) and 40 mg/kg (red) 5-HTP increases looking duration in animals with low baseline looking (dashed line) during saline sessions and decreases looking duration in high baseline looking animals (solid line). **b** (Top) Average looking duration for low and high baseline animals (grouped solid and dash lines from **a**). (Bottom) Average looking duration for low and high baseline animals after shuffling the drug labels within each animal. **c** 5-HTP significantly increases the magnitude change, the absolute value of the percent change, in looking duration relative to saline. **d** (Top) Baseline looking duration to images negatively correlates with percent change in looking duration due to 5-HTP (green, 20 mg/kg; red, 40 mg/kg). (Bottom) The average correlation from the shuffled data. In **a, c, d**, each shape represents an individual subject’s data

We used looking duration as a proxy measure to investigate how 5-HTP modulates attention to images (Fig. 1a). We first examined looking duration, for all animals, to all images, during saline, 20 mg/kg, and 40 mg/kg sessions. When all animals were analyzed together, drug dose did not impact raw looking duration (Fig. 2a, *F*(2, 287) = 1.12, *P* = 0.33). However, 5-HTP significantly increased looking duration in three subjects, those that exhibited low baseline attention during the eight saline sessions (average baseline looking of 1572.94 ± 460.85 ms), but significantly decreased looking duration in the other three subjects, those that exhibited high baseline attention during the eight saline sessions (average baseline looking of 2672.72 ± 362.16 ms, Fig. 2b). To account for this bi-directional effect, we calculated the absolute value of the percent change in looking duration from saline and found that 5-HTP significantly modulated looking duration across animals (Fig. 2c, *F(*2, 287) = 23.03, *P* < 0.01). We next investigated whether this diversity in 5-HTP’s effect on attention is related to differences in baseline attention by quantifying each subjects’ percent change from saline in looking duration due to eight sessions each of 20 mg/kg and 40 mg/kg 5-HTP. We found that baseline looking duration was negatively correlated with 5-HTP-induced changes in looking duration (Fig. 2d, *r* = −0.81, *P* < 0.01). To ensure that the observed effect was indeed due to 5-HTP differentially modulating the looking behavior across individual animals and not due to a regression to the mean phenomenon, we randomly shuffled the labels associated with each session for each animal (saline, 20 mg/kg, or 40 mg/kg 5-HTP) and re-ran the above analyses 1000 times to create a null distribution for what could be expected if 5-HTP was not truly impacting looking duration but instead was due to a regression to the mean. In these analyses, all the percent changes in looking duration were far outside the range of the observed data (*P* = 0.02, *t*-test), and the shuffled data exhibited no correlation (Fig. 2b, c lower panels, *r* = −0.14, *P* = 0.66, Pearson’s). Thus, acute 5-HTP increased looking duration in animals with low baseline attention but decreased looking duration in animals with a high baseline attention.

### Animals exhibit different levels of task engagement, but not motivation to acquire juice

We next characterized whether all animals were equally engaged in the task to determine if differences in attention to images were related to differences in task motivation, both to acquire juice and also to view images. We assessed motivation to acquire juice by quantifying the number of trials completed per session for high and low baseline attention animals. Neither baseline attention (*F*(1,138) = 2.55, *P* = 0.11, ANOVA) nor drug dose (*F*(2,143) = 0.91, *P* = 0.41) significantly affected the number of trials completed per session, indicating that animals completed the same number of trials, thereby earning the same amount of juice, regardless of baseline attention or drug dosage.

We then assessed anticipatory looking between trials and used this measure as a proxy for task engagement, that is an animal’s motivation to initiate another trial quickly and view more images [46]. We quantified anticipatory looking by calculating the percentage of trials during which subjects looked at the region near the fixation during the inter-trial interval when the screen was blank prior to the start of a new trial (See Supplementary Materials and Methods). The magnitude change in anticipatory looking was impacted by drug dose (saline, 20 mg/kg, and 40 mg/kg 5-HTP) (*F(*1, 131) = 8.93, *P* < 0.01, ANOVA). Notably, baseline anticipatory looking (during saline sessions) was negatively correlated with 5-HTP-induced changes in anticipatory looking (Fig. S1A, *r* = −0.72, *P* < 0.01). Low baseline attention animals exhibited lower task engagement than high baseline attention animals during saline sessions; 5-HTP increased engagement in low baseline animals, and decreased engagement in high baseline animals (Fig. S1B).

### Social specificity of 5-HTP’s effect is related to differences in baseline attention to social and non-social images

**Fig. 3:**
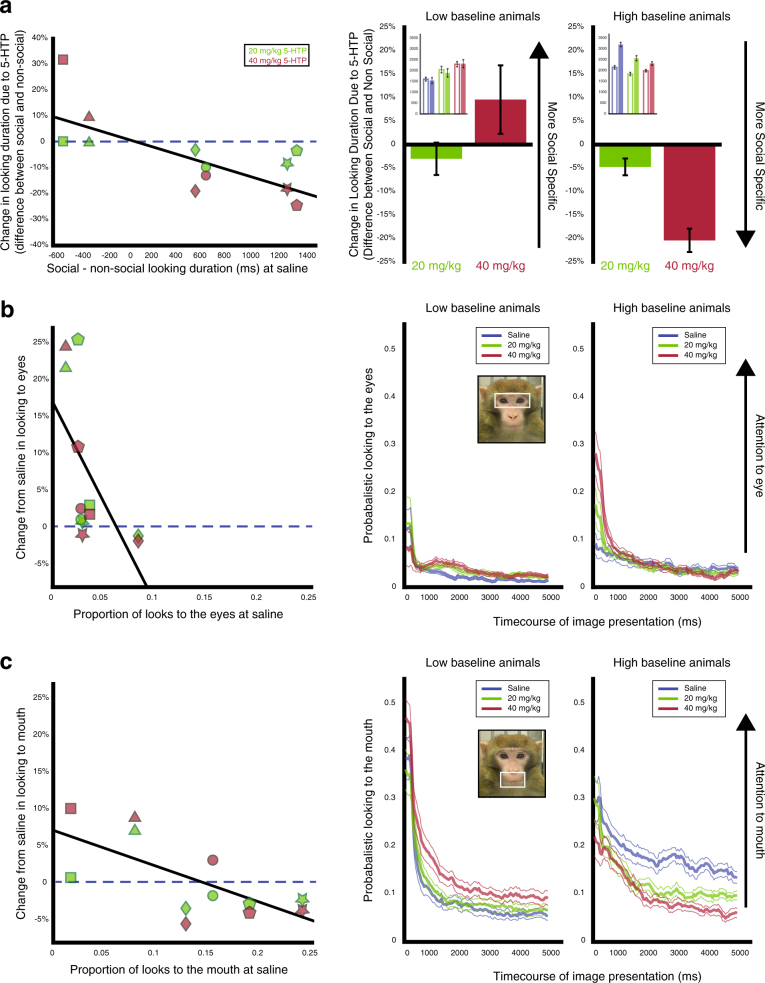
5-HTP differentially modulates attention to facial features. **a** The difference between looking duration to social and non-social images is negatively correlated with the difference in percent change from saline, due to 20 mg/kg (green) or 40 mg/kg 5-HTP (red), in looking duration to social and non-social images. (Right) The average difference in percent change in looking duration to social images and percent change in looking duration to non-social images for low and high baseline animals. The inset shows the raw looking duration to social (filled bars) and non-social images (open bars) for low and high baseline animals. **b** The percent change from saline due to 5-HTP in the probability of looking at the eye region during image presentation is negatively correlated with baseline probabilistic looking to the eye. (Right) Average time plots for low and high baseline animals for 5-HTP’s effect on attention to the eyes. **c** The percent change from saline due to 5-HTP in the probability of looking at the mouth region is negatively correlated with baseline probabilistic looking to the mouth. 5-HTP increases attention towards the mouth in low baseline animals but decreases attention towards the mouth in high baseline animals. (Right) Average time plots for low and high baseline animals for the bi-directional effect on attention to the mouth. In **a**-**c**, each shape represents an individual subject’s data

Social stimuli are inherently more salient than non-social stimuli. Animals overall looked at social images longer than non-social images (*F(*1, 287) = 15.31, *P* < 0.001, ANOVA, Fig. S2), and 5-HTP overall modulated looking to social images more than non-social images (Fig. 3a, *F(*1, 287) = 13.51, *P* < 0.001, see Fig. S3 for raw data for each subject). When we examined if variation in 5-HTP’s social specificity was predicted by baseline behavior, the difference between looking duration to social and non-social images at saline was negatively correlated with the difference in 5-HTP-induced changes in looking duration to social and non-social images (Fig. 3a, *r* = −0.72, *P* < 0.01, Pearson’s). Thus, for individuals who looked longer at social than non-social images at baseline, 5-HTP decreased looking to social more than to non-social images. By contrast, for individuals who looked longer at non-social images at baseline, 5-HTP instead increased looking to social more than to non-social images. We next investigated whether the same animals that spent less time looking at social stimuli at baseline were also the animals that exhibited baseline lower attention to all images. We found that indeed looking duration to all images were positively correlated with the average difference between looking duration to social and non-social images during saline sessions (*r* = 0.84, *P* = 0.04) indicating that animals who looked at all images for a longer period during saline sessions also exhibited a longer relative looking duration to social images during saline sessions. This provides further support that baseline differences in attention predict the manner in which 5-HTP modulates attention.

### 5-HTP bi-directionally modulates attention to the eyes and mouth

We next focused on the mouth and eye regions of the face to test if 5-HTP modulates attention to salient face regions differently depending on gaze directions (directed vs. averted) and facial expressions (threat vs. fear grimace vs. lip smack vs. neutral). Raw looking duration and percent change in looking duration due to 5-HTP for the face region of each social category are shown in the Supplementary Materials Results and Figs. S4 and S5. We calculated the percentage of trials that subjects looked within the eye or mouth regions to obtain the magnitude change, absolute value of the percent change, in probabilistic looking to the eyes and mouth due to 5-HTP. This allowed us to examine differences in 5-HTP’s effect on probabilistic looking due to stimulus monkey gaze direction and facial expression.

#### Eyes

The magnitude change in probabilistic looking to the eyes was impacted by drug dose (Fig. 3b, *F(*2, 1024) = 31.37, *P* < 0.001, ANOVA, with a stronger effect of 40 mg/kg compared to 20 mg/kg 5-HTP, *P* < 0.001, Tukey), gaze direction (directed vs. averted) (*F(*1, 1024) = 7.28, *P* < 0.01, with a larger effect for faces with direct gaze), as well as facial expression (threat vs. fear grimace vs. lip smack vs. neutral) (*F(*3, 1024) = 18.03, *P* < 0.001, larger for expressive than for neutral expressions, all *P* < 0.001). These results indicate that 5-HTP differentially influences attention to the eyes based on the gaze direction and the saliency of the facial expression of the stimulus monkeys. We next asked whether 5-HTP’s effects on attention to the eyes are related to differences in baseline attention to the eyes. Baseline looking to the eye region was negatively correlated with 5-HTP-induced changes in looking to the eye region (Fig. 3b, *r* = −0.60, *P* = 0.04, Pearson’s). 5-HTP thus increased attention to the eyes in animals with low baseline attention but decreased looking duration in animals with high baseline attention.

#### Mouth

The magnitude change from saline in probabilistic looking to the mouth was also affected by drug dose (Fig. 3c, *F(*2, 1054) = 30.53, *P* < 0.001, with a stronger effect of 40 mg/kg compared to 20 mg/kg 5-HTP, *P* < 0.001) and facial expression (threat vs. fear grimace vs. lip smack vs. neutral) (*F(*3, 1054) = 2.95, *P* = 0.03, with only fear grimaces as larger than neutral expressions after correcting for multiple comparisons, *P* = 0.02), but not gaze direction (directed vs. averted) (*F(*1, 1054) = 1.63, *P* = 0.2). These results indicate that 5-HTP differentially influenced attention to the mouth based on whether the facial expression relied on salient mouth features to communicate social signals. We again asked whether 5-HTP’s effects on attention to the mouth are related to differences in baseline attention, and found that baseline looking to the mouth was negatively correlated with 5-HTP-induced changes in looking (Fig. 3c, *r* = −0.68, *P* = 0.02). 5-HTP thus increased attention to the mouth in animals with low baseline attention but decreased looking duration in animals with high baseline attention.

Taken together, these results indicate that 5-HTP increases attention to informative regions of the face for animals with low baseline attention to these regions, but decreases attention to the eyes and mouth for animals with high baseline attention. Overall 5-HTP thus modulates attention to salient facial features that convey important social information and the direction and magnitude of 5-HTP’s effects can be predicted by baseline differences in how individuals allocate attention to the eyes and mouth.

### Baseline central serotonergic function predicts the direction and magnitude of 5-HTP’s effects on attention

**Fig. 4:**
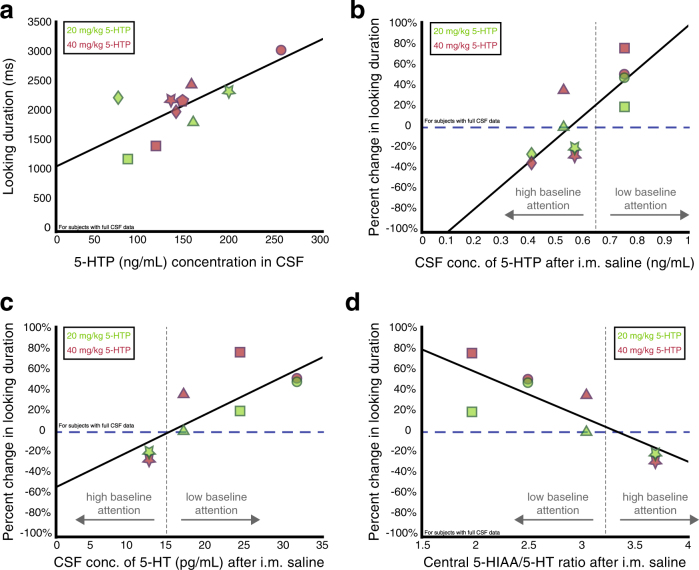
Serotonergic function predicts how 5-HTP modulates looking behavior. **a** Central concentrations of 5-HTP after receiving 20 mg/kg (green) and 40 mg/kg (red) 5-HTP injections are positively correlated with the average looking duration to social images during the 5-HTP sessions associated with each drug dose. **b** Central levels of 5-HTP at saline correlate with percent change in looking duration from saline due to 5-HTP. **c** Baseline central levels of serotonin (5-HT) correlate with the percent change in looking duration from saline due to 5-HTP. **d** Baseline central 5-HIAA/serotonin (5-HT) ratios correlate with the percent change in looking duration from saline due to 5-HTP. In **a-d**, each shape represents an individual subject’s data

To examine the relationship between central serotonergic processing and looking behavior, we first tested if the amount of 5-HTP that crossed the blood-brain barrier would predict differences in attention during 5-HTP sessions. Central concentrations of 5-HTP after receiving 20 mg/kg and 40 mg/kg injections of 5-HTP were positively correlated with individuals’ average looking durations during the sessions associated with each drug dose (Fig. 4a, *r* = 0.73, *P* = 0.016, Pearson’s correlation), suggesting that the amount by which 5-HTP injections modulate central concentrations of 5-HTP did in fact influence how long individuals looked at images.

We next determined if central concentrations of serotonergic compounds were related to 5-HTP’s observed bi-directional effects on attention by comparing 5-HTP-induced changes in attention to baseline CSF concentrations of 5-HTP and serotonin. Interestingly, baseline 5-HTP concentrations were positively correlated with the percent change in looking duration due to 5-HTP (Fig. 4b, *r* = 0.81, *P* < 0.01), suggesting that baseline levels of 5-HTP predict the manner in which attention is modulated by 5-HTP. Concentrations of baseline serotonin were also positively correlated with the percent change from saline in looking duration due to 5-HTP (Fig. 4c, *r* = 0.78, *P* = 0.02), providing more evidence that concentrations of central serotonergic compounds, even prior to 5-HTP manipulations, influence, in part, how 5-HTP will influence attention. In fact, baseline tryptophan and HVA concentrations were also correlated with the percent change in looking duration due to 5-HTP (tryptophan: *r* = 0.77, *P* = 0.01; HVA: *r* = 0.80, *P* < 0.01), while baseline 5-HIAA concentrations trended to be correlated with the percent change in looking duration (*r* = 0.62, *P* = 0.05). However, baseline concentrations of neither norepinephrine nor tyrosine were correlated with the percent change in looking duration due to 5-HTP (norepinephrine: *r* = 0.56, *P* = 0.15; tyrosine: *r* = 0.50, *P* = 0.14). These results further suggest that baseline concentrations of serotonergic related compounds are related to 5-HTPs effects on attention and that dopaminergic activity could also play a role.

Despite this converging evidence, we were puzzled that animals with low baseline attention exhibit higher baseline concentrations of 5-HTP and serotonin compared to high baseline attention animals. We conjectured that this discrepancy might be due to differences in the rate of serotonin turnover. While our experiment did not allow us to provide direct evidence, we analyzed a metric that has been used to estimate turnover in tissue samples, the 5-HIAA/serotonin ratio. CSF 5-HIAA/serotonin ratios at baseline were inversely correlated with the percent change in looking duration (Fig. 4d, *r* = −0.82, *P* = 0.03), indicating that animals with lower baseline attention might exhibit lower serotonergic turnover compared to animals with higher baseline attention. In addition, the central ratio of 5-HIAA to serotonin also correlated with average difference between looking duration to social and non-social images during saline sessions (*r* = 0.98, *P* = 0.02) indicating that a higher baseline 5-HIAA/5-HT ratio predicts a larger preference for social images over non-social images at baseline.

## Discussion

Most antidepressants inhibit the serotonin transporter, thereby increasing extracellular functionally active levels of serotonin. This observation has been used to suggest that the serotonin system plays a role in symptomatology of depression and anxiety. However, studies that have examined the causal role of serotonin in attention and emotion recognition have produced largely inconsistent results. Here, we examined how increasing central serotonin via acute administration of the precursor 5-HTP, either 20 mg/kg or 40 mg/kg, would modulate attention using a repeated, within-subject, design. Working with an animal model allowed us to examine differences in central serotonergic functioning and turnover and confirm, for the first time in non-human primates, that acute 5-HTP administration, especially the higher 40 mg/kg dose, crosses the blood-brain barrier and increases central concentrations of 5-HTP and serotonin.

While 5-HTP modulated looking duration in all subjects, 5-HTP increased looking duration in half our subjects (*n* = 3), but decreased looking duration in the other subjects (*n* = 3). Critically, baseline differences in attention predicted the direction and magnitude by which 5-HTP modulated looking duration. 5-HTP increased attention in subjects with low baseline attention yet decreased it in those with high baseline attention, and these effects were not driven by a regression to the mean (low panels of Fig. 2b, d). While most previous studies have reported a consistent modulation of emotion recognition and biased attention to expressive faces due to serotonin manipulations [11], three previous investigations reported effects of serotonin manipulation on emotional recognition and emotional biases that differed according to baseline behavior [47–49]. Bhagwagar et al. [49]. reported that a single dose of the SSRI citalopram increased the recognition of fearful faces in healthy volunteers, but decreased fear recognition in subjects with a previous history of depression [49]. Hayward et al. [47]. showed that low-dose acute tryptophan depletion (ATD) caused a decrease in the recognition of happy faces in healthy volunteers, but increased happy recognition in recovered depressed patients [47]. Finally, Robinson et al. (2010) found that baseline mood state influenced the direction in which ATD impacted cognitive biases [48].

Although 5-HTP modulated looking duration to both social and nonsocial images, we observed a greater effect of 5-HTP on attention to social images. Intriguingly, this social specificity was again linked to baseline differences in attention to social and non-social images, providing additional evidence that 5-HTP’s effects on attention can be predicted by differences in baseline behavior. Previous literature indicates that SSRIs increase recognition of, and attention to, positive and negative facial expressions, but the field remains uncertain whether SSRIs act via a general mechanism or through separate negative and positive bias mechanisms in tandem [11, 50, 51]. In addition, most emotional processing studies in humans do not include non-social controls [11]. Based on our results, it is likely that serotonin generally modulates attention, and that the specificity of its effects in the social domain likely depends on baseline differences in how individuals attend to these stimuli.

5-HTP particularly increased attention to salient facial features that convey important social information, the eyes and mouth, in animals with low baseline attention, but decreased attention to these same regions in animals with high baseline attention. Reducing serotonergic function via ATD in humans decreases sensitivity towards emotional faces when they display directed gaze towards participants, but not when they exhibit averted gaze [52]. 5-HTP may modulate attention to facial regions as a function of how salient they are and also how useful they are to decode signal content. Perhaps this is the mechanism by which serotonin acts when modulating competent behavior, particularly impulsive aggression and dominance status, as reported in previous work [20, 53–58].

Differences in serotonergic function may underlie 5-HTP’s diverse effects across individuals. While differences in central concentrations of 5-HTP and serotonin at baseline positively predicted the direction in which, and magnitude by which, 5-HTP modulated looking duration, CSF analyses confirmed that 5-HTP administration increased central concentrations of 5-HTP and serotonin, and caused pupil constriction in all animals, indicating a consistent physiological effect of 5-HTP on the central nervous system and a dissociation of the parasympathetic nervous system from the serotonin-mediated control of attention allocation. However, we did not expect that low baseline attention animals would exhibit higher baseline concentrations of 5-HTP and serotonin than high baseline attention animals. It could be that animals’ serotonergic systems differ not only according to active concentrations of 5-HTP and serotonin, but also in terms of the efficiency of the serotonergic system. Although we did not directly test the serotonergic turnover rate at the biochemical level in tissue samples, we investigated the relationship between percent change in looking duration and ratio of 5-HIAA to serotonin in CSF. Generally, based on prior studies in tissue samples, higher central 5-HIAA to serotonin ratios indicate an increased turnover of serotonin as it is converted to 5-HIAA within the brain [5]. While this ratio is only an estimate of this rate, we found that the 5-HIAA to serotonin ratio predicted both looking duration at baseline and also the percent change in looking duration due to 5-HTP. Overall, animals with low baseline attention exhibited lower baseline 5-HIAA to serotonin ratios, attended to images less at baseline and exhibited more positive changes in looking duration due to 5-HTP than their high baseline attention counterparts.

Our findings provide unique causal and mechanistic evidence suggesting that enhancing central serotonergic function results in categorically distinct changes in fundamental cognitive operations such as attention. Future work replicating these findings with more animals could further clarify individual differences in attention and central serotonergic concentrations. Amongst our sample of six monkeys, there were no consistent differences in age, dominance status, or health of any animals. Detecting any such potential relationships would require a larger sample size. In addition, continued efforts towards better understanding the relationship between behavioral and biochemical phenotypes and the behavioral effects of serotonergic manipulations could contribute to improved prediction of clinical outcome of serotonergic treatment in numerous psychiatric conditions.

## Electronic supplementary material

Supplementary Materials

Supplementary Figure 1

Supplementary Figure 2

Supplementary Figure 3

Supplementary Figure 4

Supplementary Figure 5

Supplementary Table 1

Supplementary Table 2

Supplementary Table 3
