# Supplementary material for: The effects of 5-hydroxytryptophan on attention and central serotonin neurochemistry in the rhesus macaque
Source: Neuropsychopharmacology. 2018 Jan 30;43(7):1589–98. doi: 10.1038/s41386-017-0003-7 (PMC5983545; doi:10.1038/s41386-017-0003-7)
Supplement: Supplementary file 1 — Supplementary Materials [file 41386_2017_3_MOESM1_ESM.docx]

**Supplementary Materials**

The Effects of 5-Hydroxytryptophan on Attention and Central Serotonin Neurochemistry in the Rhesus Macaque

Hannah Weinberg-Wolf^1^, Nicholas A. Fagan^1^, George M. Anderson^2^, Marios Tringides^1^, Olga Dal Monte^1^ and Steve W. C. Chang^1,2,3,4^

1: Department of Psychology, Yale University, New Haven, CT 06520

2: Child Study Center, Yale University School of Medicine, New Haven, CT 06510

3: Department of Neuroscience, Yale University School of Medicine, New Haven, CT 06510

4: Kavli Institute for Neuroscience, Yale University School of Medicine, New Haven, CT 06510

Included are:

**Supplementary Results**

**Supplementary Discussion**

**Supplementary Materials and Methods**

**Supplementary Figure**

**Supplementary References**

**Supplementary Results**

**Exogenous 5-HTP does not increase CSF concentrations of** **5-HIAA, homovanillic acid (HVA), tryptophan, tyrosine, or norepinephrine.**

We first tested if 5-HTP administrations increased central concentrations of 5-HIAA, HVA, tryptophan, tyrosine, and norepinephrine. Subjects’ CSF was sampled at one hour after an injection of saline, 20mg/kg 5-HTP and 40mg/kg 5-HTP. Drug injections did not affect central 5-HIAA concentrations (F(2,3)=1.29, P=0.33, ANOVA), HVA concentrations (F(2,3)=1.20, P=0.36, ANOVA), tryptophan (F(2,3)=1.72, P=0.25, ANOVA), tyrosine (F(2,3)=1.24, P=0.35, ANOVA), or norepinephrine (F(2,3)=0.37, P=0.72, ANOVA).

**Central concentrations of 5-HIAA, homovanillic acid (HVA), tryptophan, tyrosine, or norepinephrine do not predict 5-HTP’s effects on attention.**

To determine if central concentrations of serotonergic compounds were related to 5-HTP’s bi-directional effects on attention, we compared behavioral changes to saline CSF concentrations of 5-HIAA, tryptophan, tyrosine, and norepinephrine. We found that baseline tryptophan and HVA concentrations were correlated with the percent change in looking duration due to 5-HTP (tryptophan: r= 0.77, P=0.01; HVA: r= 0.80, P<0.01), while baseline 5-HIAA concentrations trended to be correlated with the percent change in looking duration (r= 0.62, P=0.05). However, baseline concentrations of neither norepinephrine nor tyrosine were correlated with the percent change in looking duration due to 5-HTP (norepinephrine: r= 0.56, P=0.15; tyrosine: r= 0.50, P=0.14). This suggests that baseline concentrations of serotonergic related compounds might be related to 5-HTPs effects on attention and that dopaminergic activity could play a role as well.

**Effects of 5-HTP on Facial Expression and Gaze Direction**

Macaques rely on stereotyped facial expressions to communicate. In our task, macaques viewed faces of conspecifics exhibiting neutral expressions, open-mouth threat expressions, fear-grimace submissive expressions, and affiliative lip-smack expressions with either directed or averted gaze ([de Waal and Luttrell, 1985](#_ENREF_12); [Maestripieri, 1997](#_ENREF_17); [Maestripieri and Wallen, 1997](#_ENREF_18); [Partan, 2002](#_ENREF_19)). Given that rhesus macaques rely on facial expression and gaze direction to evaluate social information, we investigated whether 5-HTP manipulated looking duration to social images differently depending on the expressions (threat vs. fear grimace vs. lip smack vs. neutral) and gaze direction (directed vs. averted) conveyed by stimulus monkeys. We found that while drug dose (saline, 20mg/kg 5-HTP, and 40mg/kg 5-HTP) impacted the magnitude change in looking duration to social images (*F(*2, 1150)=78.14, *P*<0.001, Figs. S2, S3), neither facial expression (*F(*3, 1150)=0.81, *P*=0.50, Figs. S2, S3) nor gaze direction (*F(*1, 1504)=1.00 *P*=0.32, Figs. S2, S3) impacted the magnitude change in looking duration to social images due to 5-HTP.

**Genetic Variation Results**

Extensive work has indicated that variation in three genes related to the production and transportation of serotonin is associated with decreased serotonergic function. Furthermore, these minor alleles have been extensively implicated in impaired social attention ([Beevers et al, 2010](#_ENREF_7); [Beevers et al, 2011](#_ENREF_8); [Pérez-Edgar et al, 2010](#_ENREF_21)), increased impulsive aggression ([Dobson and Brent, 2013](#_ENREF_13)), social anxiety ([Hariri and Holmes, 2006](#_ENREF_15)), as well as a variety of impaired social abilities ([Canli and Lesch, 2007](#_ENREF_10)). All six subjects were genotyped for these three alleles: the polymorphic region of the serotonin transporter (5-HTTLPR), tryptophan hydroxylase 2 (TPH-2), and monoamine oxidase A (MAO-A) ([Watson et al, 2009](#_ENREF_23)). All subjects were dominant homozygote for the rh5-HTT. For the rh-MAOA, four subjects displayed the six repeat allele, one subject displayed the five repeat allele and one subject displayed the seven repeat allele. Matching our hypothesis, the individual with the rh7 fell into the low baseline looking group and the individual with the rh5 fell into the high baseline looking group. Mechanistically this is logical as lower MAOA expression corresponds to less serotonin degradation. As a result, input of serotonin into the system could result in longer effects of serotonin. Five subjects displayed dominant homozygote alleles for the rh-TPH2 polymorphism. However, one subject was heterozygous long/short. Again matching our expected hypothesis, the presence of a long allele mapped onto an individual that displayed low initial looking behavior. Long alleles are linked to lower social interest so it is again logical to see the individual expressing the recessive allele displaying a reduced attention prior to addition to serotonin. While, these results cannot be interpreted statistically due to the small sample size, it is interesting that those who exhibited genetic variants had the expected behavior associated with those variants.

**Supplementary Discussion**

**Effects of 5-HTP on Salient Facial Features**

The looking behavior effects of 5-HTP were larger for images with conspecific faces conveying directed gaze and non-neutral expressions. In rhesus macaques, threat faces signal dominance, fear-grimaces signal context dependent subordinance, and lip-smacks are used to affiliate and diffuse conflict ([de Waal *et al*, 1985](#_ENREF_12); [Maestripieri, 1997](#_ENREF_17); [Maestripieri *et al*, 1997](#_ENREF_18); [Partan, 2002](#_ENREF_19)). The mouth is generally considered the most salient facial feature, especially when images are presented statically, that macaques use to differentiate these expressions ([Partan, 2002](#_ENREF_19); [Waller and Micheletta, 2013](#_ENREF_22)). In addition, previous work has shown that macaques saccade to the eye region of directed faces earlier and for longer than averted faces ([Leonard *et al*, 2012](#_ENREF_16)), exhibit gaze following behavior from infancy, and have evolved circuitry to follow the social and non-social gaze of conspecifics ([Emery, 2000](#_ENREF_14)). Cells within the macaque amygdala, orbitofrontal cortex, and superior temporal sulcus are specifically tuned to follow the gaze of conspecifics ([Allison *et al*, 2000](#_ENREF_1)) and play a role in shifting gaze to conspecifics ([Chang *et al*, 2015](#_ENREF_11)).

**Consideration for Future Work**

Future work should take steps to investigate how differences in receptor density and receptor binding relate to differences in central serotonergic turnover rate and also investigate how this variation relates to differences in baseline attention. While we were able to collect many sessions of data from each animal, due to the invasive nature of CSF draws, we were limited to, at most, one sample of CSF per animal per drug dose, and CSF was collected on different days than behavioral measures. Future work replicating our findings with more animals could further clarify individual differences in attention and central serotonergic concentrations. Amongst our sample of 6 monkeys, there were no consistent differences in age, dominance status, or health of any animals. Detecting any such potential relationships would require a larger sample size.

**Supplementary Materials and Methods**

**Surgery**

Before testing began, subjects received a surgically implanted head-restraining device to allow for accurate video tracking of eye movements. At the time of surgery, anesthesia was induced with ketamine hydrochloride (10 mg/kg i.m.) and maintained with isoflurane (1.0-3.0%, to effect). Subjects received isotonic fluids via an intravenous drip. Aseptic procedures were employed. Heart rate, respiration rate, blood pressure, expired CO_2_, and body temperature were monitored throughout the procedure. After the head restraining device implantation was completed, the wound around the base was closed in anatomical layers. Subjects received a peri- and post-operative treatment regimen consisting of 0.01 mg/kg buprenorphine every 12 hours for 3 days, 0.1 mg/kg meloxicam once daily for 3 days, and 5 mg/kg baytril once daily for 10 days. Subjects were allowed 40+ days of recovery after the implant surgery before training began and were slowly acclimated to head restraint over a week of training.

**CSF Analysis Protocols**

Samples with gross blood contamination (>0.1%), as indicated by pink coloration, were excluded prior to screening for hemoglobin. Limiting blood contamination to <0.1% was sufficient to ensure that analyses other than serotonin were not affected by blood. However, all CSF samples analyzed for serotonin were screened more rigorously for blood contamination by measuring hemoglobin using Multistix 8 SG reagent strips for urinalysis (Bayer Corp., Elkhart, IN), which can detect approximately 0.2 μg/ml of hemoglobin. As previously demonstrated, screening for hemoglobin and using only those samples with <10 ppm blood limited blood-derived serotonin in CSF to less than 10 pg/ml ([Anderson *et al*, 2005](#_ENREF_2)). Neurochemical analyses levels of CSF 5-HTP, serotonin, 5-HIAA, homovanillic acid (HVA), tryptophan, tyrosine, and norepinephrine were determined using reverse-phase high performance liquid chromatography (HPLC) as previously described ([Anderson *et al*, 2002](#_ENREF_3); [Anderson *et al*, 1987a](#_ENREF_4); [Anderson *et al*, 1990](#_ENREF_5); [Anderson *et al*, 1987b](#_ENREF_6)).

Samples with any detectable blood contamination were excluded from serotonin and norepinephrine analysis – a total of 4 samples were excluded. The final 5-HTP CSF data therefore included samples collected after injection of saline, 20mg/kg, and 40mg/kg 5-HTP from 4 subjects with an additional subject contributing samples at saline and 40mg/kg and a final subject contributing samples at 40mg/kg. The final serotonin CSF data set was more restricted and includes saline data from 4 subjects, 20mg/kg 5-HTP data from 3 subjects, and 40mg/kg data from 4 subjects.

**Additional Information on Experimental Design**

The subjects sat in a primate chair (Precision Engineering Co.) 47 cm away from the screen. MATLAB (Math Works) with Psychtoolbox ([Brainard, 1997](#_ENREF_9)) and Eyelinktoolbox ([Pelli, 1997](#_ENREF_20)) was used to display stimuli and collect eye position data. Horizontal and vertical eye positions were sampled at 1,000 Hz using an infrared eye monitor camera system (SR Research Eyelink). Monkeys first acquired and held a central fixation square for 300 ms to receive a 0.1 mL bridge juice reward. After the bridge reward, either a social (a conspecific face) or a non-social stimulus (scrambled face, or landscape scene) was displayed centrally for 5,000 ms. Subjects received a larger, 0.3 mL, juice reward at stimulus offset regardless of the image type or of how long the subjects looked at the stimuli (Fig. 1A). A solenoid valve controlled the delivery of the fluid reward.

Valid trials were defined as those in which the monkeys successfully held fixation for 300 ms during the pre-image fixation interval. If monkeys broke the fixation, the trial was aborted, no image appeared, and the animal was not rewarded and instead received a 1,500 ms timeout. We included all successful trials even if the animals did not look at the image displayed after the initial fixation was completed.

Our main behavioral measure of interest was looking duration, expressed as the total time the monkey spent looking at an image. Valid trials were defined as those in which the monkeys successfully held fixation for 300 ms during the pre-image fixation interval. If monkeys broke fixation, the trial was aborted, no image appeared, and the animal was not rewarded and instead received a 1,500 ms timeout. We included all successful trials even if the animals did not look at the image displayed after the initial fixation was completed. We excluded trials from the analysis that were more than two standard deviations away from the mean looking duration within dose and image category. Using this criterion we excluded only 3% of our trials.

**Data Analyses**

To directly examine whether 5-HTP modulated looking duration to images, we calculated the total time animals spent looking at social and non-social images. Data were averaged within sessions. Looking duration was assessed using a 3x2 ANOVA model specifying drug dose (saline, 20mg/kg 5-HTP, and 40mg/kg 5-HTP) and image category (social versus non social) as fixed factors. To account for the fact that 5-HTP *increased* looking duration in 3 animals, but *decreased* looking duration in 3 animals (Figure 2A), we quantified the magnitude of the change in looking duration due to 5-HTP. For each session we calculated the absolute value of the percent change in looking duration from the average of all saline sessions. We ran a 2x3 ANOVA model specifying image category (social versus non social) and drug dose (saline, 20mg/kg 5-HTP, and 40mg/kg 5-HTP) as fixed factors. All sessions of the saline data were included in our model and normalized to the average of saline; this conservative approach accounts for variability in looking duration during saline sessions. Direct post hoc comparisons were made with two tailed independent *t*-tests and *P*-values were corrected for multiple comparisons with a Tukey test. Correlations were reported by calculating a Pearson's linear correlation coefficient.

We also asked if 5-HTP modulated looking duration differently depending on stimulus monkeys’ facial expressions and gaze directions by examining magnitude change due to 5-HTP for each social image category. Session averaged data was assessed using a 3x2x4 ANOVA model specifying drug dose (saline, 20mg/kg 5-HTP, and 40mg/kg 5-HTP), stimulus face gaze direction (directed vs. averted), and stimulus monkey expression (threat vs. fear grimace vs. lip smack vs. neutral). This analysis allowed us to determine if 5-HTP differentially modulated looking duration to images based on facial expression and gaze direction. Direct post hoc comparisons were made with two tailed independent *t*-tests and the *P*-value was corrected for multiple comparisons with a Tukey test.

To examine individual’s attention to the eyes and mouth, we calculated the percentage of trials that subjects looked within the eye or mouth regions during each sliding window (non-overlapping 50 ms bins) throughout the image presentation (5,000 ms) during saline sessions. To calculate the amount of time subjects looked at the eye and mouth regions of social images, one researcher custom created a rectangular region of interest for the eyes and mouth separately on each image while another researcher confirmed all regions. The coordinates of each region were maintained in a custom MATLAB script and used to determine when subjects looked within these regions on each stimulus. This allowed us to compensate for systemic size differences in the eye and mouth based on expression and gaze direction without reducing the ecological validity afforded by using large numbers of unedited images. The total time the subjects spent looking within the boundaries of the image, eye, and mouth were calculated using a custom script written in MATLAB. We included all successful trials (see above) but required at least one fixation to the image. We averaged across the entire time window to calculate the mean probabilistic looking to each the eyes and mouth. We then quantified the absolute value of the percent change in probabilistic looking to the eyes and mouth due to 5-HTP, for 20mg/kg and 40mg/kg, to account for 5-HTP’s bi-directional effects. This allowed us to examine differences in 5-HTP’s effect on probabilistic looking to the eyes and mouth due to stimulus monkey gaze direction and facial expression. Data were averaged within sessions, and the magnitude change in probabilistic looking were assessed using two separate 3x2x4 ANOVA models, one for the eyes and one for the mouth, each specifying drug dose (saline, 20mg/kg, and 40mg/kg 5-HTP), stimulus face gaze direction (directed vs. averted), and stimulus monkey expression (threat vs. fear grimace vs. lip smack vs. neutral). Direct post hoc comparisons were made with two tailed independent *t*-tests and the *P*-value was corrected for multiple comparisons with a Tukey test. The correlations between probabilistic looking to the eyes or mouth at saline and percent change due to 5-HTP were reported by calculating a Pearson's linear correlation coefficient.

To quantify anticipatory looking, we calculated the percentage of trials during which subjects looked at the region of the screen where the new fixation stimulus would later in time appear within each sliding window (non-overlapping 50 ms bins) throughout the inter-trial interval (ITI, 1500 ms). We averaged across this time window to determine a mean probabilistic looking. Once again, we accounted for 5-HTP’s bi-directional effects by calculating the absolute value of the percent change in anticipatory looking due to 5-HTP. Data were averaged within sessions, and differences in the absolute value of the percent change in anticipatory looking were assessed using a one way ANOVA model specifying drug dose (saline, 20mg/kg 5-HTP, and 40mg/kg 5-HTP). Direct post hoc comparisons were made with two tailed independent *t*-tests and the *P*-value was corrected for multiple comparisons with a Tukey test. The correlation between anticipatory looking at saline and percent change in anticipatory looking due to 5-HTP was reported by calculating a Pearson's linear correlation coefficient.

Changes in CSF concentrations and pupil size were assessed using repeated measures ANOVA models specifying drug dose (saline, 20mg/kg 5-HTP, and 40mg/kg 5-HTP) as a fixed factor. For each of these analyses, we carried out direct post hoc comparisons with two tailed independent *t*-tests and corrected the *P*-value for multiple comparisons with a Tukey test.

Finally, correlations between percent change in looking duration from saline, raw looking duration, and CSF concentrations were reported by calculating a Pearson's linear correlation coefficient.

**Supplementary Figures and Legends**

**Figure S1: 5-HTP di-directionally modulates task engagement. A)** The probability, expressed as the percentage of trials, that animals look at the region near the fixation during the inter-trial-interval. Baseline anticipatory looking is negatively correlated with the percent change in anticipatory looking due to 20mg/kg (green) and 40mg/kg (red) 5-HTP. Each shape represents an individual subject’s data. **B)** Average time plots for low and high baseline animals to illustrate 5-HTP’s bi-directional effect on anticipatory looking. (green, 20 mg/kg; red, 40 mg/kg; blue, saline).

**Figure S2: Raw looking to conspecific faces, versus scrambled faces and landscape images.** Looking duration from saline sessions is shown in blue, 20mg/kg 5-HTP sessions in green, and 40mg/kg 5-HTP sessions in red. Data is plotted per subject. **A)** Low baseline looking animals. **B)** High baseline looking animals. Soc. stands for social images, Scr. stands for luminance matched scrambled images, Land. stands for landscape images. Each shape corresponds to the data of a single subject.

**Figure S3: Percent change in looking duration to conspecific faces, versus scrambled faces and landscape images due to 5-HTP.** Labeling conventions are the same as in Figure S2.

**Figure S4: Raw looking duration to each social image category.** Looking duration from saline sessions is shown in blue, 20mg/kg 5-HTP sessions in green, and 40mg/kg 5-HTP sessions in red. Data is plotted per subject. **A)** Low baseline looking animals. **B)** High baseline looking animals. T refers to faces exhibiting threat expressions, S refers to faces exhibiting fear grimaces, L refers to faces exhibiting lip smacks, and N refers to faces exhibiting neutral expressions. Each shape corresponds to the data of a single subject.

**Figure S5: Percent change in looking duration to each social image category due to 5-HTP.** Labeling conventions are the same as in Figure S4.

**Supplementary Tables**

**Supplemental Table 1:** Pair-wise correlation of CSF concentrations of monoamines, their precursors, and metabolites, for all data points. Significant correlations are bolded while trending to significant correlations are in italics.

**Supplemental Table 2:** Pair-wise correlation of CSF concentrations of monoamines, their precursors, and metabolites, but only for data collected after i.m. 20 mg/kg 5-HTP and 40 mg/kg 5-HTP. Same significance notations as Table 1.

**Supplemental Table 3:** Pair-wise correlation of CSF concentrations of monoamines, their precursors, and metabolites, but only for data collected after i.m. saline. Same significance notations as Table 1.

Uncategorized References

Allison T, Puce A, McCarthy G (2000). Social perception from visual cues: role of the STS region. *Trends in cognitive sciences* **4**(7): 267-278.

Anderson GM, Barr CS, Lindell S, Durham AC, Shifrovich I, Higley JD (2005). Time course of the effects of the serotonin-selective reuptake inhibitor sertraline on central and peripheral serotonin neurochemistry in the rhesus monkey. *Psychopharmacology* **178**(2-3): 339-346.

Anderson GM, Bennett AJ, Weld KP, Pushkas JG, Ocame DM, Higley DJ (2002). Without Title. *Psychopharmacology* **161**(1): 95-99.

Anderson GM, Feibel FC, Cohen DJ (1987a). Determination of serotonin in whole blood, platelet-rich plasma, platelet-poor plasma and plasma ultrafiltrate. *Life sciences* **40**(11): 1063-1070.

Anderson GM, Mefford IN, Tolliver TJ, Riddle MA, Ocame DM, Leckman JF*, et al* (1990). Serotonin in human lumbar cerebrospinal fluid: a reassessment. *Life sciences* **46**(4): 247-255.

Anderson GM, Teff KL, Young SN (1987b). Serotonin in cisternal cerebrospinal fluid of the rat: measurement and use as an index of functionally active serotonin. *Life sciences* **40**(23): 2253-2260.

Beevers CG, Ellis AJ, Wells TT, McGeary JE (2010). Serotonin transporter gene promoter region polymorphism and selective processing of emotional images. *Biological psychology* **83**(3): 260-265.

Beevers CG, Marti CN, Lee H-J, Stote DL, Ferrell RE, Hariri AR*, et al* (2011). Associations between serotonin transporter gene promoter region (5-HTTLPR) polymorphism and gaze bias for emotional information. *Journal of Abnormal Psychology* **120**(1): 187.

Brainard DH (1997). The psychophysics toolbox. *Spatial vision* **10**: 433-436.

Canli T, Lesch K-P (2007). Long story short: the serotonin transporter in emotion regulation and social cognition. *Nature neuroscience* **10**(9): 1103-1109.

Chang SW, Fagan NA, Toda K, Utevsky AV, Pearson JM, Platt ML (2015). Neural mechanisms of social decision-making in the primate amygdala. *Proceedings of the National Academy of Sciences* **112**(52): 16012-16017.

de Waal F, Luttrell LM (1985). The formal hierarchy of rhesus macaques: an investigation of the bared‐teeth display. *American Journal of Primatology* **9**(2): 73-85.

Dobson SD, Brent LJ (2013). On the evolution of the serotonin transporter linked polymorphic region (5-HTTLPR) in primates. *Frontiers in human neuroscience* **7**: 588.

Emery NJ (2000). The eyes have it: the neuroethology, function and evolution of social gaze. *Neuroscience & Biobehavioral Reviews* **24**(6): 581-604.

Hariri AR, Holmes A (2006). Genetics of emotional regulation: the role of the serotonin transporter in neural function. *Trends in cognitive sciences* **10**(4): 182-191.

Leonard TK, Blumenthal G, Gothard KM, Hoffman KL (2012). How macaques view familiarity and gaze in conspecific faces. *Behavioral neuroscience* **126**(6): 781.

Maestripieri D (1997). Gestural communication in macaques: usage and meaning of nonvocal signals. *Evolution of communication* **1**(2): 193-222.

Maestripieri D, Wallen K (1997). Affiliative and submissive communication in rhesus macaques. *Primates* **38**(2): 127-138.

Partan SR (2002). Single and multichannel signal composition: facial expressions and vocalizations of rhesus macaques (Macaca mulatta). *Behaviour* **139**(8): 993-1027.

Pelli DG (1997). The VideoToolbox software for visual psychophysics: Transforming numbers into movies. *Spatial vision* **10**(4): 437-442.

Pérez-Edgar K, Bar-Haim Y, McDermott JM, Gorodetsky E, Hodgkinson CA, Goldman D*, et al* (2010). Variations in the serotonin-transporter gene are associated with attention bias patterns to positive and negative emotion faces. *Biological psychology* **83**(3): 269-271.

Waller BM, Micheletta J (2013). Facial expression in nonhuman animals. *Emotion Review* **5**(1): 54-59.

Watson KK, Ghodasra JH, Platt ML (2009). Serotonin transporter genotype modulates social reward and punishment in rhesus macaques. *PloS one* **4**(1): e4156.
