# Supplementary figures and images for: The effects of 5-hydroxytryptophan on attention and central serotonin neurochemistry in the rhesus macaque
Source: Neuropsychopharmacology. 2018 Jan 30;43(7):1589–98. doi: 10.1038/s41386-017-0003-7 (PMC5983545; doi:10.1038/s41386-017-0003-7)

**a**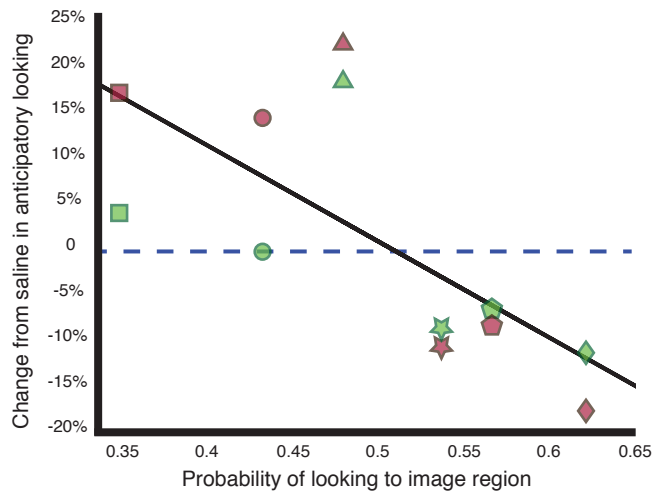**b**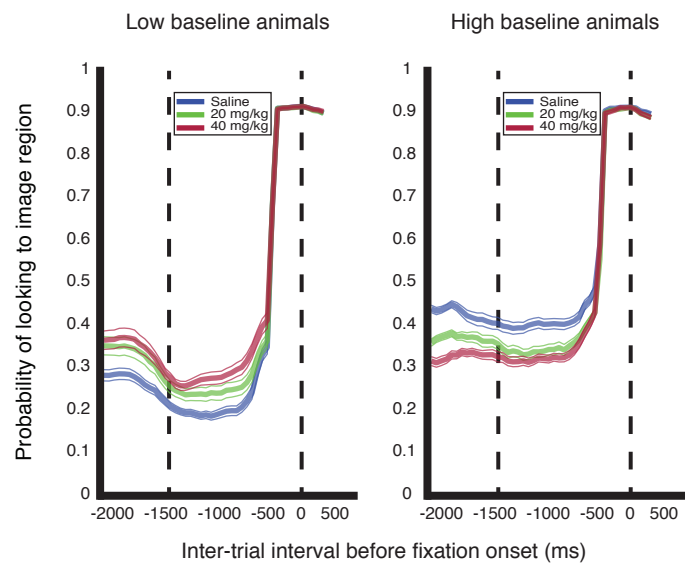

Supplement: Supplementary file 2 — Supplementary Figure 1 [file 41386_2017_3_MOESM2_ESM.pdf]
