# Supplementary material for: The effects of 5-hydroxytryptophan on attention and central serotonin neurochemistry in the rhesus macaque
Source: Neuropsychopharmacology. 2018 Jan 30;43(7):1589–98. doi: 10.1038/s41386-017-0003-7 (PMC5983545; doi:10.1038/s41386-017-0003-7)
Supplement: Supplementary file 3 — Supplementary Figure 2 [file 41386_2017_3_MOESM3_ESM.pdf]

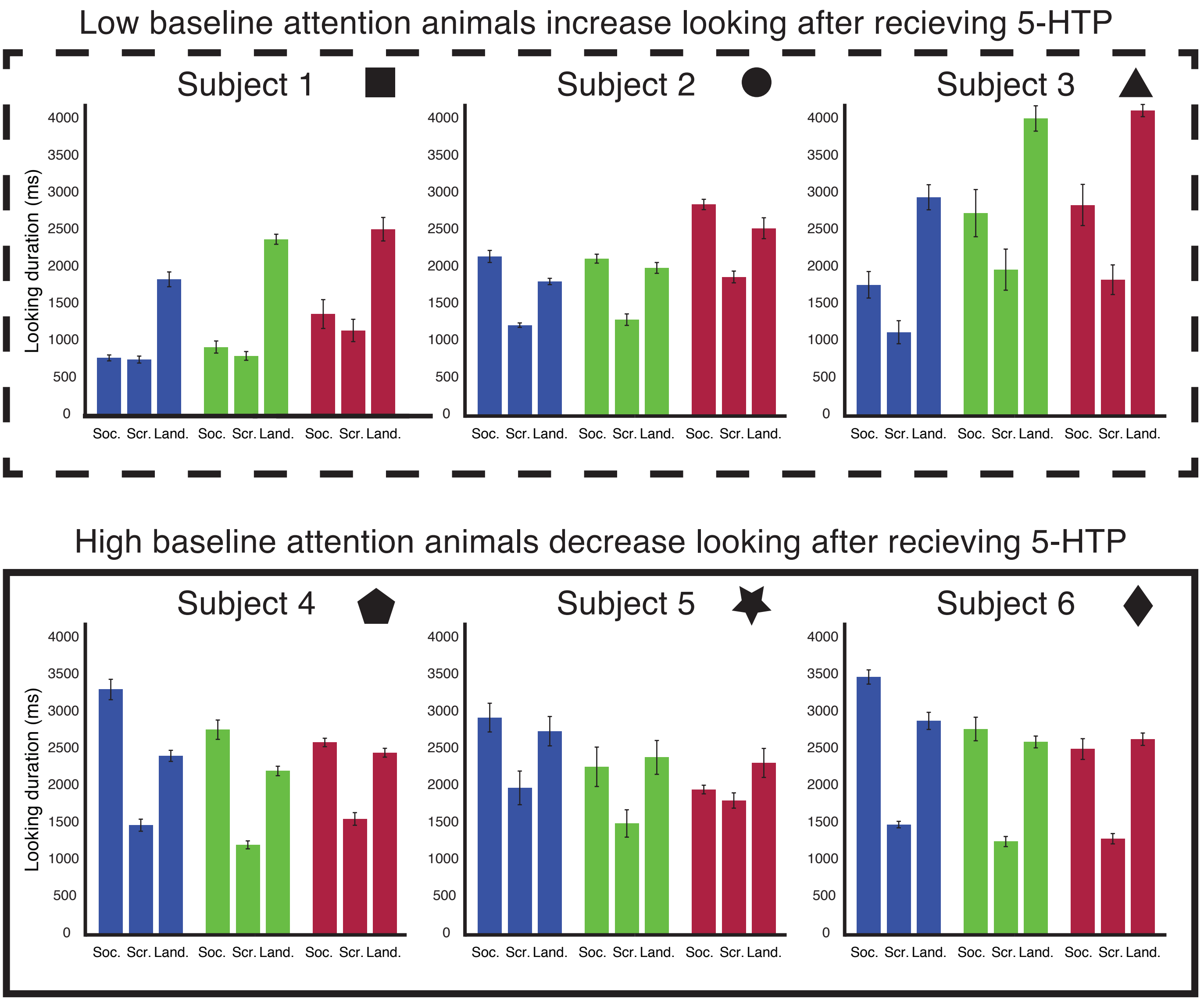
Low baseline attention animals increase looking after recieving 5-HTP
Subject 1
Subject 2
Subject 3
Looking duration (ms)
0
500
1000
1500
2000
2500
3000
3500
4000
Soc. Scr. Land. Soc. Scr. Land. Soc. Scr. Land.
High baseline attention animals decrease looking after recieving 5-HTP
Subject 4
Subject 5
Subject 6
