# Supplementary material for: The effects of 5-hydroxytryptophan on attention and central serotonin neurochemistry in the rhesus macaque
Source: Neuropsychopharmacology. 2018 Jan 30;43(7):1589–98. doi: 10.1038/s41386-017-0003-7 (PMC5983545; doi:10.1038/s41386-017-0003-7)
Supplement: Supplementary file 6 — Supplementary Figure 5 [file 41386_2017_3_MOESM6_ESM.pdf]

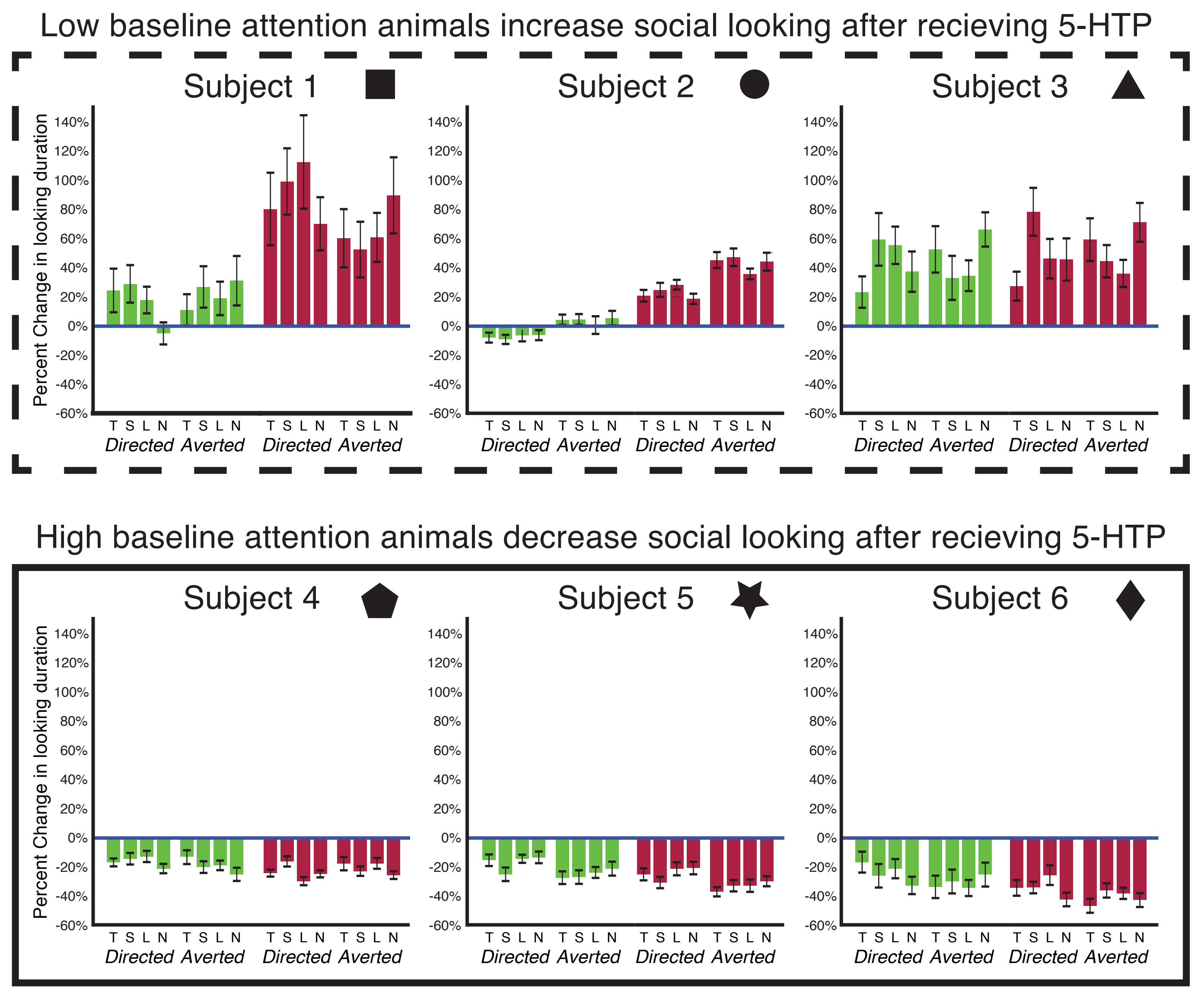
Low baseline attention animals increase social looking after recieving 5-HTP
Subject 1
Subject 2
Subject 3
High baseline attention animals decrease social looking after recieving 5-HTP
Subject 4
Subject 5
Subject 6
Percent Change in looking duration
140%
120%
100%
80%
60%
40%
20%
0%
-20%
-40%
-60%
T S L N T S L N T S L N T S L N
Directed Averted Directed Averted
