# Supplementary material for: The effects of 5-hydroxytryptophan on attention and central serotonin neurochemistry in the rhesus macaque
Source: Neuropsychopharmacology. 2018 Jan 30;43(7):1589–98. doi: 10.1038/s41386-017-0003-7 (PMC5983545; doi:10.1038/s41386-017-0003-7)
Supplement: Supplementary file 7 — Supplementary Table 1 [file 41386_2017_3_MOESM7_ESM.pdf]

Pair-wise correlation of CSF concentrations for all data points

	HVA	5-HIAA	5-HT	5-HTP	TRP	TYR	NE
**HVA**		**r= 0.72 P< 0.01**	r= -0.34 P= 0.31	r= 0.04 P= 0.90	**r= 0.69 P< 0.01**	r= 0.7 P= 0.80	r= 0.14 P= 0.68
**5-HIAA**			r= 0.24 P= 0.48	**r= 0.55 P= 0.03**	**r= 0.63 P= 0.01**	r= -0.23 P= 0.41	r= 0.26 P= 0.44
**5-HT**				**r= 0.78 P< 0.01**	r= -0.27 P= 0.43	**r= -0.64 P= 0.03**	r= 0.12 P= 0.73
**5-HTP**					r= -0.06 P= 0.83	r= -0.37 P= 0.17	r= 0.01 P= 0.98
**TRP**						r= -0.16 P= 0.58	r= 0.43 P= 0.18
**TYR**							r= -0.11 P= 0.75
**NE**							
