# Supplementary material for: The effects of 5-hydroxytryptophan on attention and central serotonin neurochemistry in the rhesus macaque
Source: Neuropsychopharmacology. 2018 Jan 30;43(7):1589–98. doi: 10.1038/s41386-017-0003-7 (PMC5983545; doi:10.1038/s41386-017-0003-7)
Supplement: Supplementary file 8 — Supplementary Table 2 [file 41386_2017_3_MOESM8_ESM.pdf]

Pair-wise correlation of CSF concentrations for data collected after i.m. 5-HTP

	HVA	5-HIAA	5-HT	5-HTP	TRP	TYR	NE
**HVA**		**r= 0.75 P= 0.01**	r= -0.08 P= 0.86	*r= 0.59 P= 0.07*	r= 0.50 P= 0.14	r= -0.15 P= 0.68	r= -0.62 P= 0.14
**5-HIAA**			r= 0.29 P= 0.53	r= 0.93 P< 0.01	r= 0.67 P= 0.03	r= -0.40 P= 0.25	r= -0.24 P= 0.60
**5-HT**				r= 0.41 P= 0.36	r= 0.09 P= 0.85	r= -0.41 P= 0.35	r= 0.03 P= 0.95
**5-HTP**					*r= 0.63 P= 0.05*	r= -0.49 P= 0.15	r= 0.01 P= 0.99
**TRP**						*r= -0.60 P= 0.06*	r= -0.06 P= 0.89
**TYR**							r= -0.46 P= 0.30
**NE**							
