# Supplementary material for: The effects of 5-hydroxytryptophan on attention and central serotonin neurochemistry in the rhesus macaque
Source: Neuropsychopharmacology. 2018 Jan 30;43(7):1589–98. doi: 10.1038/s41386-017-0003-7 (PMC5983545; doi:10.1038/s41386-017-0003-7)
Supplement: Supplementary file 9 — Supplementary Table 3 [file 41386_2017_3_MOESM9_ESM.pdf]

Pair-wise correlation of CSF concentrations for data collected after i.m. saline

	HVA	5-HIAA	5-HT	5-HTP	TRP	TYR	NE
**HVA**		**r= 0.92 P= 0.03**	**r= 0.99 P< 0.01**	*r= 0.85 P= 0.07*	**r= 0.99 P< 0.01**	r= 0.55 P= 0.30	**r= 0.96 P= 0.04**
**5-HIAA**			r= 0.83 P= 0.17	r= 0.66 P= 0.22	**r= 0.92 P= 0.03**	r= 0.59 P= 0.30	**r= 0.96 P= 0.04**
**5-HT**				r= 0.87 P= 0.13	**r= 0.97 P= 0.03**	r= 0.23 P= 0.77	*r= 0.91 P= 0.09*
**5-HTP**					*r= 0.87 P= 0.05*	r= 0.75 P= 0.14	r= 0.73 P= 0.27
**TRP**						r= 0.61 P= 0.27	**r= 0.98 P= 0.02**
**TYR**							r= 0.56 P= 0.44
**NE**							
